# Sensitivity of Noninvasive Prenatal Detection of Fetal Aneuploidy from Maternal Plasma Using Shotgun Sequencing Is Limited Only by Counting Statistics

**DOI:** 10.1371/journal.pone.0010439

**Published:** 2010-05-03

**Authors:** H. Christina Fan, Stephen R. Quake

**Affiliations:** Department of Bioengineering, Stanford University and Howard Hughes Medical Institute, Stanford, California, United States of America; Brunel University, United Kingdom

## Abstract

We recently demonstrated noninvasive detection of fetal aneuploidy by shotgun sequencing cell-free DNA in maternal plasma using next-generation high throughput sequencer. However, GC bias introduced by the sequencer placed a practical limit on the sensitivity of aneuploidy detection. In this study, we describe a method to computationally remove GC bias in short read sequencing data by applying weight to each sequenced read based on local genomic GC content. We show that sensitivity is limited only by counting statistics and that sensitivity can be increased to arbitrary precision in sample containing arbitrarily small fraction of fetal DNA simply by sequencing more DNA molecules. High throughput shotgun sequencing of maternal plasma DNA should therefore enable noninvasive diagnosis of any type of fetal aneuploidy.

## Introduction

Current diagnosis of fetal aneuploidy is based on karyotyping fetal cells obtained from amniocentesis or chorionic villus sampling. These invasive procedures can potentially result in miscarriages [1]. The discovery of fetal cells and cell-free fetal nucleic acids in maternal blood in the past few decades have prompted researchers to seek for methods to diagnose fetal aneuploidy noninvasively. However, the small amount of fetal genetic material relative to the maternal counterparts in maternal blood poses a technical challenge for such task [2].

We recently achieved noninvasive detection of fetal aneuploidy [3]. The technique uses high-throughput shotgun sequencing of maternal plasma cell-free DNA, of which a small fraction originates from the fetus, followed by mapping short sequence tags to the chromosome of origin. The ability to count millions of DNA sequences allowed us to detect small changes in the representation of chromosomes contributed by an aneuploid fetus in a maternal plasma sample. We successfully detected over-representation of chromosomes 13, 18, and 21 in the respective cases trisomy 13, 18, and 21 pregnancies, and under-representation of chromosome X in male pregnancies. Our results were quickly reproduced by an independent group [4], [5], which used a slightly different statistical approach in determining the representation of chromosomes. In principle, both techniques should reveal deviation of copy number from normal for any chromosome given sufficient amount of counting. However, both our results and those of the independent group deviated from ideal counting statistics. Such deviation, caused largely by GC bias in the sequencing data, placed a practical limit on the sensitivity of the test especially for detecting aneuploidy associated with chromosomes that are GC-poor or GC-rich [3]. Our observations indicated that GC bias among samples was more substantial between sequencing runs than within runs, suggesting that such bias might have been introduced during the sequencing process, perhaps through slight differences in reagent composition and temperatures that led to differences in DNA denaturation and primer hybridization efficiency.

Here, we describe a method to computationally remove GC bias in short read sequencing data and reanalyze the data from our previous work. This treatment of the data eliminates most variance in the distribution of sequence tag counts across the genome and enables the detection of fetal aneuploidy with greater statistical confidence. The current sensitivity is limited only by counting statistics, and therefore it should be possible to increase the sensitivity of noninvasive detection of any type of fetal aneuploidy to arbitrary precision for maternal sample containing arbitrarily small fetal DNA fraction, simply by sequencing more molecules. Since there are measurable amounts of fetal DNA in maternal plasma as early as day 18 after embryo transfer by assisted reproduction [6], this suggests that it may be possible to perform noninvasive prenatal diagnosis for fetal aneuploidy as early as desired in the pregnancy, and potentially at the first doctor's visit.

## Methods

### Data

The data analyzed in this study was collected previously [3]. It consisted of short (25 nucleotides) sequence tags obtained by shotgun sequencing cell-free plasma DNA collected from 19 pregnant women on the Illumina/Solexa platform.

### Removal of the effect of GC bias in the density of sequence tags

We first investigated the relation between GC content of the sequenced DNA fragments and the number of sequence tag. We suspected that GC bias was introduced during PCR in library preparation and cluster generation in the Illumina/Solexa sequencing workflow. Because the entire DNA fragment (content between the two adaptor sequences) but not only the 25 sequenced bases participated in the PCR while the exact length of each sequenced fragment was not known, we chose to calculate GC content of 20 kb non-overlapping bins across the entire human genome. Since the human genome is made up of isochores, which are >300 kb regions of relatively homogeneous GC content [7], sequenced fragments lying within the isochore would most likely have GC content similar to the isochore. Thus, any regions much smaller than 300 kb, including the choice of 20 kb in our analysis, should provide a rough estimate of the GC content of each sequenced fragment. Such calculation was performed using the *hgGcPercent* script of the UCSC Genome Browser's kent source tree. The output file contained the coordinate of each 20 kb bin and the corresponding GC content.

**Figure 1:**
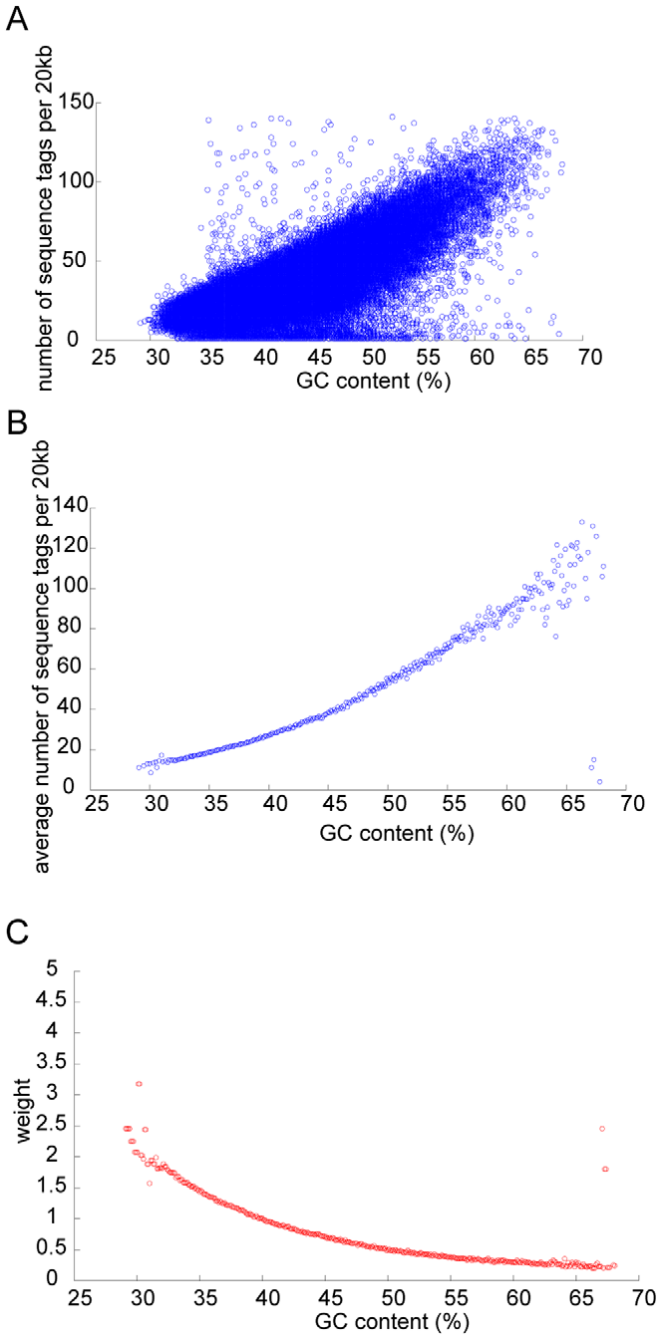
Illustration of the procedures used to remove GC content dependent artifact in shotgun sequencing data using one of the patient samples as example. (A) The number of sequence tag per 20 kb bin is plotted against GC content of the bin. (B) The average number of sequence tag per 20 kb is calculated for every 0.1% GC content. (C) A weight is calculated for a particular value of GC content, such that sequence tags falling within a 20 kb bin having such GC content would receive the same calculated weight.

For each sample, the number of sequence tag in each 20 kb bin was counted. The average number of sequence tag per 20 kb bin, *M_i_*, was calculated for every 0.1% GC content, ignoring bins with no reads, bins with zero GC percent, and bins with over-abundant reads. All sequence tags falling within a 20 kb bin with GC content *i* were assigned weights 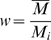, where 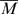 is the global average number of sequence tag per 20 kb. Our strategy was based on the assumption that the sequence tags would be uniformly distributed across the genome had there been no influence of GC content. Figure 1 illustrates the procedure for one of the patient samples. The weighted number of sequence tag within every 50 kb non-overlapping window was then summed to obtain the distribution of sequence tag density for each chromosome. The choice of 50 kb was arbitrary but was used in our previous work.

A baseline was constructed by calculating the following expression for the *k^th^* 50 kb bin in the *i^th^* chromosome: 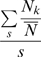, where *N_k_* is the number of sequence tag in the *k^th^* bin, 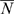 is the average number of sequence tag per bin in the entire genome, and *s* is the number of patient samples carrying two copies of chromosome *i*. The final sequence tag distributions were obtained by normalizing the GC corrected distribution by the baseline.

### Detection of fetal aneuploidy

To determine whether the copy number of a chromosome within a patient sample deviated from normal, the sequence tag density (i.e. number of sequence tag per 50 kb bin) distribution of a chromosome was compared to all other chromosomes (except chromosome Y). The null hypothesis was that two distributions had equal mean number of sequence tags per 50 kb, while the alternative hypothesis was that the means were unequal. The *z*-statistic, 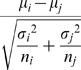 was calculated, where *μ* is the mean sequence tag density; *σ* is the standard deviation of the distribution of sequence tag density, and *n* is the number of bins, and subscript *i, j* represents chromosomes *i, j*. Each chromosome has a different size, thereby affecting its sensitivity of detection; the varying number of bins *n* for each chromosome takes this effect into account. We required that a chromosome to be significantly different from all other chromosomes at level *α*<0.001 (two-tailed) to be called abnormal. We employed Bonferroni correction to take into account the 22×23 multiple comparisons within each sample (i.e. |*z*|> 4.756 for the null hypothesis to be rejected).

### Estimation of sequencing depth requirement for fetal aneuploidy detection

For a given fetal DNA fraction *ε*, we could estimate the sequencing depth required in order to detect deviation of chromosome copy number from normal at some level of confidence α. When comparing the distributions of sequence tag densities for any two chromosomes *i* and *j*, their means would be significantly different if 
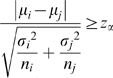
, where *μ* is the mean sequence tag density; *σ* is the standard deviation of the distribution of sequence tag density, *n_i_* is the number of bins for chromosome *i* (chromosome of interest), *n_j_* is the number of bins for chromosome *j* (*i≠j*), and *z_α_* is the *z-*statistic associated with the level of confidence α. If chromosome *i* is abnormal and chromosome *j* is normal in terms of copy number, the above expression can be approximated as 
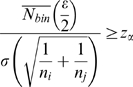
, where 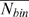 is the genome-wide average of number of sequence tags within a 50 kb bin; and 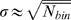 assuming normal approximation of the Poisson distribution. Since 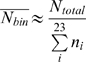, where *n_23_* denotes the number of 50 kb bins for chromosome X, the minimum total number of reads required (i.e. sequencing depth), *N_total_*, for each sample can be estimated as 
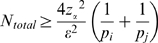
, where *p_i_* is the proportion of chromosome *i* in terms of size relative to the entire genome, or 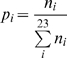. Because our analyses of experimental data required that the sequence tag distribution of a chromosome to be significantly different from all other chromosomes to be called abnormal, in our estimation for *N_total_*, *n_j_* was the number of bins of the shortest autosomes, which was either chromosome 21 or 22.

### Estimation of fraction of fetal DNA

The fraction of fetal DNA in a cell-free DNA sample can sometimes be estimated from the final sequence tag distributions. Since chromosome X is under-represented in male pregnancies, the fetal DNA fraction was estimated as 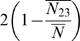, where 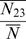 is the average number of sequence tag per bin for chromosome X normalized to the global average. The fetal DNA fraction was estimated as 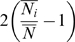, where 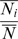 is the normalized average number of sequence tag per bin for chromosome 13, 18, or 21 for the aneuploid samples.

## Results

**Figure 2:**
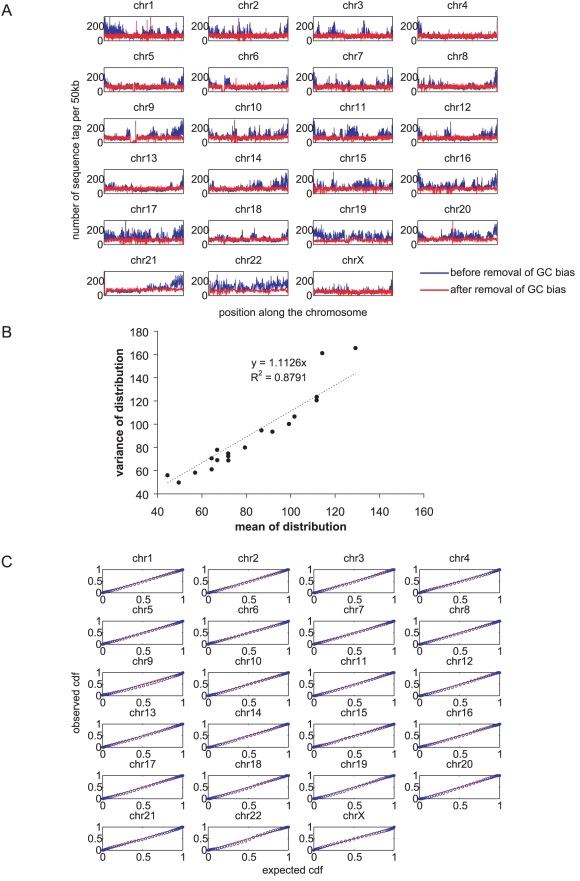
Removal of GC bias reveals that sampling of sequence tags closely follows the Poisson distribution. (A) Sequence tag distribution (number of sequence tags per 50 kb bin) across each chromosome before (blue) and after (red) applying GC dependent weighting to sequence tags, using one patient sample as example. (B) Plot of variance against mean of sequence tag density for each chromosome in every sample. (C) Comparing the cumulative distribution of the sequence tag density against that predicted by the Poisson distribution using one patient sample as example. The red diagonal line has a slope of 1 and an intercept of 0.

Because the degree of GC bias was different in different sample, each sample had its own weighting scheme. Figure 2A shows the distribution of sequence tags across each chromosome before and after correction for GC bias for one of the 19 samples. Applying weighting to sequence tags depending on local GC percentage eliminated most variations in the distribution of sequence tags across each chromosome. Through examinations of the GC corrected sequence tag distributions of all patient samples, we observed consistent patterns of region specific variations. We further removed these variations by dividing the GC corrected sequence tag distribution of each sample by that averaged from all samples. For any sample, if the regions with unusually large number of sequence tags (most probably due to repeats) were ignored (by removing 50 kb regions with greater or fewer than 1.96*standard deviation of the chromosome specific sequence tag density), the ratio of the variance and the mean of the distribution of sequence tag density for any chromosome in any sample was close to 1, indicating that the sampling of sequences fundamentally followed Poisson statistics (Figure 2B). This observation was strengthened by comparing the cumulative distribution of sequence tag density of each chromosome with that predicted by the Poisson distribution (Figure 2C).

**Figure 3:**
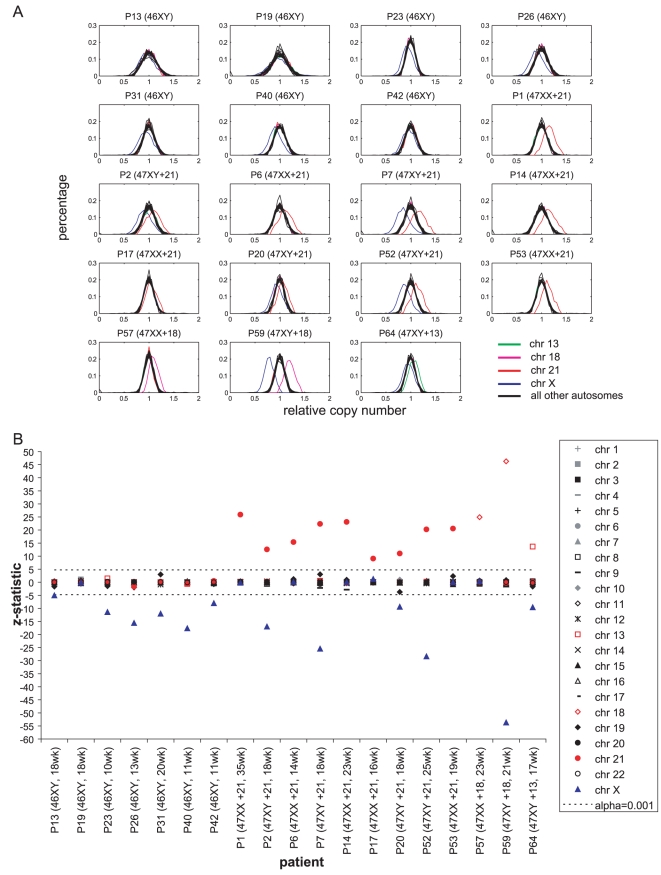
Comparing the distribution of sequence tag density of each chromosome among 19 patients after correcting for GC bias. (A) Distributions of normalized sequence tag density for each chromosome (excluding chromosome Y) within each patient sample. Red: chromosome 21; blue: chromosome X; magenta: chromosome 18; green: chromosome 13; black: all other autosomes. For all but one (P19) male pregnancy sample, it is obvious that the distribution of chromosome X shifts towards the left, while the distributions of chromosomes 21, 18, and 13 for the respective cases of trisomy 21, 18, and 13 shift towards the right, relative to the distributions of all other chromosomes that are present in two copies. (B) The sequence tag distribution of each chromosome is compared to all other chromosomes (except chromosome Y) by calculating the *z*-statistic. If we require that the copy number of a chromosome to be significantly different from that of all other chromosomes at level *α*<0.001 to be flagged as abnormal, chromosome X is under-represented as compared to a normal female genome in all but one male pregnancy (P19), while chromosomes 21, 18, and 13 are over-represented in the respective cases of trisomy 21, 18, and 13. Plotted here is the minimum *z*-statistic for each chromosome when it is compared against 22 other chromosomes. The horizontal dashed line corresponds to the statistic associated with *α*<0.001.

Figure 3A contains plots of the distribution of normalized sequence tag density for each chromosome (excluding chromosome Y) within each patient sample. For all but one male pregnancy sample, it was obvious that the distribution of chromosome X shifted towards the left, while the distributions of chromosomes 21, 18, and 13 for the respective cases of trisomy 21, 18, and 13 shifted towards the right, relative to the distributions of all other chromosomes that were present in two copies. If we required that the copy number of a chromosome to be significantly different from that of all other chromosomes at level *α*<0.001 to be flagged as abnormal, chromosome X was under-represented as compared to a normal female genome in all but one male pregnancy, while chromosomes 21, 18, and 13 were over-represented in the respective cases of trisomy 21, 18, and 13 (Figure 3B). None of the other chromosomes in the sample set were flagged.

**Figure 4:**
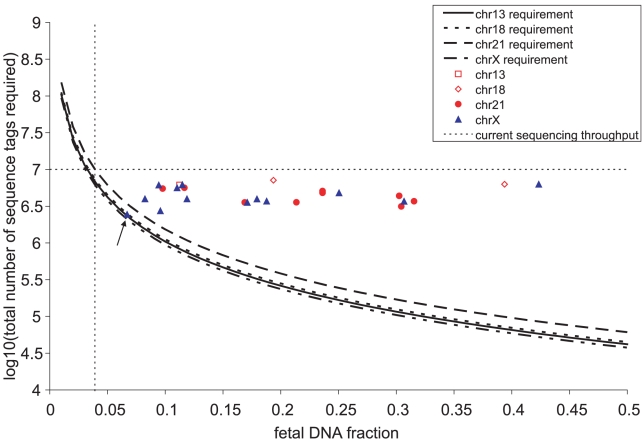
Estimation of the requirement of sequencing depth for the detection of fetal aneuploidy in cell-free plasma as a function of fetal DNA fraction. The estimates are based on level of confidence α<0.001 for chromosomes 13, 18, 21, and X, each having different length. As fetal DNA fraction decreases, the total number of shotgun sequences required increases. With a sequencing throughput of ∼10 million sequence reads per channel on the flowcell, trisomy 21 can be detected if >3.9% of the cell-free DNA is fetal (dashed lines). The total number of sequence tags and the estimated fetal DNA fraction from our set of 19 patient samples are also plotted. For one of the normal male samples (P19, indicated by the solid arrow), chromosome X was not detected as under-represented. This was probably due to insufficient sampling, as the total number of sequence obtained for this sample was close to the limit of detection given its fetal DNA fraction.

Since the removal of sequencing artifacts revealed that the sampling of sequences followed the Poisson distribution, we could estimate the total number of sequence tags (i.e the depth of sequencing) required to detect over- or under- representation of any chromosome at certain level of confidence given the fraction of fetal DNA in the maternal plasma sample (Figure 4). We also plotted the total weighted number of useful sequence tags and the estimated fetal DNA fraction for each patient sample. For male aneuploid cases, the estimates of fetal DNA fraction could be obtained from chromosome X and the trisomic chromosome, and usually were very close to each other (Table 1). We observed that the single male pregnancy sample whose chromosome X was not flagged as abnormal had a total number of sequence tags close to our estimated limit for detection at level *α*<0.001. Thus the inability to detect under-representation of chromosome X in that sample was most probably due to insufficient sampling. At the present throughput of about ∼10 million useful reads per channel on the Illumina/Solexa platform, we predict that data from a single channel enables detection of fetal trisomy 21 at *α*<0.001 in a sample containing >3.9% fetal DNA and at *α*<0.01 in a sample containing >3.5% fetal DNA.

**Table 1:** Fetal DNA fraction estimated from under-representation of chromosome X in male pregnancies and over-representation of chromosomes 13, 18, and 21 from the respective cases of trisomy 13, 18 and 21.

Patient Sample	Fetal DNA Fraction estimated from			
	chr13	chr18	chr21	chrX
P13 (46XY, 18 wk)	-	-	-	0.10
P19 (46XY, 18 wk)	-	-	-	0.07
P23 (46XY, 10 wk)	-	-	-	0.11
P26 (46XY, 13 wk)	-	-	-	0.19
P31 (46XY, 20 wk)	-	-	-	0.12
P40 (46XY, 11 wk)	-	-	-	0.18
P42 (46XY, 11 wk)	-	-	-	0.08
P1 (47XX +21, 35 wk)	-	-	0.30	-
P2 (47XY +21, 18 wk)	-	-	0.17	0.17
P6 (47XX +21, 14 wk)	-	-	0.21	-
P7 (47XY +21, 18 wk)	-	-	0.32	0.31
P14 (47XX +21, 23 wk)	-	-	0.30	-
P17 (47XX +21, 16 wk)	-	-	0.10	-
P20 (47XY +21, 18 wk)	-	-	0.12	0.11
P52 (47XY +21, 25 wk)	-	-	0.24	0.25
P53 (47XX +21, 19 wk)	-	-	0.24	-
P57 (47XX +18, 23 wk)	-	0.19	-	-
P59 (47XY +18, 21 wk)	-	0.39	-	0.42
P64 (47XY +13, 17 wk)	0.11	-	-	0.09

## Discussion

In this communication, we demonstrate an algorithm to compensate for GC bias in high-throughput short read sequencing data appropriate for the noninvasive detection of fetal aneuploidy from maternal plasma cell-free DNA. Others have shown the existence of substantial GC bias in Illumina/Solexa and ABI/SOLiD sequencing [5], [8], [9], [10], and this limits the sensitivity of the detection of under- or over-representation of chromosomes. Our method of removing GC bias in sequencing data reveals that the sampling of cell-free DNA by shotgun sequencing fundamentally obeys the statistics of counting. This implies that the difference in representation among chromosomes within a sample, however small, can be detected to arbitrary precision by sequencing deeply enough. The precise definition of “deeply enough” is that the number of DNA molecules counted be larger than the statistical noise, which scales as the square root of the sampling rate. Therefore, the only factor that limits the sensitivity is the sequencing depth. Shotgun sequencing should be able to detect fetal aneuploidy associated not only with chromosomes 21, 18, and 13 as shown here, but all other chromosomes as well. Rarer forms of aneuploidy, such as partial trisomy and monosomy, should in principle be detected using such approach provided that there is sufficient sampling. The sensitivity would depend on the length of the region of interest, sequencing depth, and fetal DNA fraction. For instance, by extending our analysis of Figure 4, we predicted that a region of ∼2.2 Mb of trisomy or monosomy would be detected given the sequencing throughput of 10 million and 10% fetal DNA. As fetal DNA fraction varies in different pregnancies and in different stages of a pregnancy [11], clinical studies would be needed to determine the minimum fetal DNA fraction and thus the general sequencing depth required. With the rapid advances in sequencing technologies, deep sampling can soon be achieved at low cost. We therefore believe that high-throughput sequencing would eventually become a nonvinasive diagnostic test for fetal aneuploidy.
